# *Porphyromonas gingivalis*, a periodontitis causing bacterium, induces memory impairment and age-dependent neuroinflammation in mice

**DOI:** 10.1186/s12979-017-0110-7

**Published:** 2018-01-30

**Authors:** Ye Ding, Jingyi Ren, Hongqiang Yu, Weixian Yu, Yanmin Zhou

**Affiliations:** 1 Department of Implantology, School and Hospital of Stomatology, Jilin University, Qinghua Road 1500, Chaoyang District, Changchun, 130021 China; 2 Key laboratory of Mechanism of Tooth Development and Jaw Bone Remodeling and Regeneration in Jilin Province, Qinghua Road 1500, Chaoyang District, Changchun, 130021 China

**Keywords:** *Porphyromonas gingivalis* (*P. gingivalis*), Periodontitis, Cognition, Neuroinflammation, Alzheimer’s disease(AD)

## Abstract

**Background:**

A possible relationship between periodontitis and Alzheimer’s disease (AD) has been reported. However, there is limited information on the association between the *Porphyromonas gingivalis* (*P. gingivalis*) periodontal infection and the pathological features of AD. The hypothesis that *P. gingivalis* periodontal infection may cause cognitive impairment via age-dependent neuroinflammation was tested.

**Results:**

Thirty 4-week-old (young) female C57BL/6 J mice were randomly divided into two groups, the control group and the experimental group. Thirty 12-month-old (middle-aged) were grouped as above. The mouth of the mice in the experimental group was infected with *P. gingivalis*. Morris water maze(MWM) was performed to assess the learning and memory ability of mice after 6 weeks. Moreover, the expression levels of the pro-inflammatory cytokines TNF-α, IL-6, and IL-1β in the mice brain tissues were determined by Quantitative real-time polymerase chain reaction (qRT-PCR), Enzyme Linked Immunosorbent Assay(ELISA) and immunohistochemistry. Our results showed that the learning and memory abilities of the middle-aged *P. gingivalis* infected mice were impaired. Moreover, the expression levels of the pro-inflammatory cytokines TNF-α, IL-6, and IL-1β in the brain tissues of the middle-aged *P. gingivalis* infected mice were increased.

**Conclusions:**

These results suggest that *P. gingivalis* periodontal infection may cause cognitive impairment via the release of the pro-inflammatory cytokines TNF-α, IL-6, and IL-1β in the brain tissues of middle-aged mice.

**Electronic supplementary material:**

The online version of this article (10.1186/s12979-017-0110-7) contains supplementary material, which is available to authorized users.

## Background

Periodontium is composed of gingiva, periodontal ligament, alveolar bone and cementum. Infection of the periodontium, known as periodontitis, is a chronic peripheral inflammatory disease, initiated by microbes residing in the oral cavity. It is commonly caused by specific bacteria, such as *P. gingivalis*, a Gram-negative bacterium, which is a key periodontal pathogen. *P. gingivalis* and its toxic components, including fimbria, gingipains, and lipopolysaccharide (LPS), are closely related to periodontitis. Clinically, chronic periodontitis is characterized by the presence of gingival erythema, edema, periodontal pockets, and destruction of the tissue supporting the teeth [1–3]. Periodontal microorganisms and their products may enter into the circulation leading to bacteremia and systemic dissemination of bacterial products [4]. Moreover, periodontitis can induce systemic effects by promoting the expression of inflammatory mediators such as pro-inflammatory cytokines. Thus, periodontitis has been confirmed to be associated with systemic diseases including cardiovascular disease, diabetes, atherosclerotic, and respiratory diseases [5].

In recent years, AD, well known as a progressive neurodegenerative disease, has been recognized as the leading cause of cognitive and behavioral damage [6]. It has been increasingly claimed that peripheral infections could activate already primed microglial cells within the central nervous system (CNS) which promotes the development of neurodegeneration in AD [7]. The mechanism by which peripheral pro-inflammatory molecules might increase the brain’s molecular inflammatory pool involves at least two pathways, the systemic circulation and/or the neural pathways. Once in the brain, pro-inflammatory molecules might directly elevate the expression levels of the pro-inflammatory cytokine pool locally, or indirectly activate glial cells by regulating the secretion of additional pro-inflammatory cytokines. Therefore, Kamer et al. first proposed that the pool of the inflammatory molecules in the brain could be enhanced by periodontitis, which is characterized by elevated inflammatory levels, and consequently promoting the development of AD [5]. Recently, there are increasing studies supporting this hypothesis. A close relationship between immunological mediators, such as TNF-α and antibodies against periodontal pathogens, and AD, has been reported. Furthermore, these mediators can be used as AD diagnostic factors [8]. In addition, a report stated that poor dentition is associated with cognitive impairment [9]. A positive correlation between cognitive decline in AD patients and both acute and chronic inflammation was revealed by a human trial [10]. Moreover, a study conducted by Sophie et al. reported that LPS from periodontal pathogens could gain access to the brain tissue of AD patients during life, demonstrating the vital role of inflammatory factors in the pathology of AD [11]. Therefore, these reports suggest a possible connection between periodontitis and AD. However, most of the previous studies have not demonstrated a clear causative relationship between periodontitis and cognitive impairment. The experimental model used in this study was more representative of periodontitis infection as it utilizes the entire *P. gingivalis* in order to take into consideration all the bacteria components and secreted compounds which might be contributing to the development of periodontitis and might also affect cognitive memory. Therefore, in this study we test our hypothesis that periodontitis may cause cognitive impairment via age-dependent neuroinflammation in the *P. gingivalis* infection animal model.

## Methods

### Animal model

All animal experiments protocols were approved by the Animal Ethics Committee of Jilin University Medical Centre (Jilin, China). Thirty 4-week-old (young) and thirty 12-month-old (middle-aged) female C57BL/6 J mice (Animal Experiment Center of Jilin University) were respectively maintained with autoclaved food, water and bedding. Fifteen young and fifteen middle-aged mice were allocated to the *P. gingivalis* infection group. The remaining mice composed the control group. Mice were treated prior to infection with kanamycin (1 mg/ml water) for 7 days to suppress resident flora growth. Mice were infected with live *P. gingivalis* ATCC33277 by oral gavage using feeding needles. The periodontitis group received 0.1 ml of *P. gingivalis* (10^9^ CFU/ml) in 2% carboxymethyl cellulose (CMC), once every 48 h, and this process repeated for the following 6 weeks. The control group received 0.1 ml of phosphate buffer saline (PBS) in 2% CMC. Mice were sacrificed 6 weeks after the first infection.

### Behavioral evaluation

MWM tests were carried out as described by M.K. et al. with few modifications [12]. MWM is used in behavioral neuroscience to study spatial learning and memory [13]. The water pool was 1.2 m in diameter and 50 cm deep. The pool was filled with nontoxic white paint opaque water. The temperature was held constant at 23 ± 1 °C. The pool was separated into four equal quadrants. A platform (10 cm^2^) laid 1 cm below the level of the surface of the water, in the center of one of these four quadrants, was considered the target quadrant. During the training days, the location of the platform remained the same. The mice were given four tests a day for four days. During the successive four days, mice were allowed to flee to hidden platforms. In the fourth trial, each experiment used a different starting point. If the mouse couldn’t find the hidden platform in 60 s, it was gently guided to the platform, and allowed to stay there for 20 s. The time taken to reach the platform (escape latency) was recorded. On the fifth day, the platform was removed from the pool. During the probe test, the mice were freed into the pool for 120 s. The amount of times the mice crossed the area where the platform had been placed were calculated by a visual tracing system (XR-XM101, Chengdu Techman Software Co. LTD).

### Immunohistochemistry

The immunohistochemical staining was performed as described by Li et al. with slight modifications [14]. Mice were sacrificed under deep anesthesia and the brains were removed. After fixing in 4% paraformaldehyde overnight at 4 °C, the brains were embedded in paraffin and then cut coronally at a thickness of 5 μm. The sections for staining were deparaffinized and washed. Following, they were heated in 0.01 M sodium citrate buffer (pH 6.0) for antigen retrieval. Endogenous peroxide activity was quenched with 3% hydrogen peroxide. Sections were incubated with primary antibodies against TNF-α (Abcam, ab6671, 1:100), IL-6 (Bioss, bs-0379R, 1:100), IL-1β (Bioss, bs-0812R, 1:100) for 1 h at 37 °C, followed by Polymer Helper and anti-rabbit IgG polymer (ZSGB-BIO, PV-9001). Finally, color was developed with 3,3-diaminobenzidine DAB (ZSGB-BIO, ZLI-9018), and then it was counterstained with hematoxylin. Images were captured using the Olympus cellSens Dimension–Experimental systems in combination with light microscopy (Olympus BX 51, Japan).

### qRT-PCR analysis

The total RNA was extracted with Trizol reagent (Invitrogen), according to the manufacturer’s instructions. A total of 1000 ng of extracted RNA was reverse transcribed to cDNA using the PrimeScript RT reagent Kit with gDNA Eraser (TaKaRa DRRO47A). 25 ng of cDNA was used to perform qRT-PCR on the QPCR Mx3005P system (Agilent Technologies Stratagene) in a final volume of 25 μl using the SYBR Premix Ex Taq II (TaKaRa RR420Q). The data were evaluated using the MxPro-Mx3005P QPCR software program. For data normalization, mouse β-actin was used to control for the cDNA input, and the relative units were calculated by a comparative Ct method. All experiments were repeated three times.

The primer sequences used for qRT-PCR are as follows:

IL-1β: 5′- TCCAGGATGAGGACATGAGCAC-3’ and 5’-GAACGTCACACACCAGCAGGTTA-3′;

IL-6: 5’-CCACTTCACAAGTCGGAGGCTTA-3’ and 5’-CCAGTTTGGTAGCATCCATCATTTC-3′;

TNF-α:5’-ACTCCAGGCGGTGCCTATGT-3’ and 5′- GTGAGGGTCTGGGCCATAGAA-3′;

β-actin:5’-CATCCGTAAAGACCTCTAGCCAAC-3’ and 5’-ATGGAGCCACCGATCCACA-3′.

### ELISA

The ELISA was carried out as described by Xu Wu et al. with few modifications [15]. The mice were sacrificed by decollation. The brain tissues were quickly removed and the cerebral cortex was carefully isolated with microscopic forceps. The isolated tissues were homogenized in PBS (pH 7.4) and then centrifuged. The supernatant was recovered and used as the test sample. The levels of TNF-α, IL-6, and IL-1β were determined by the ELISA kit (Lengton Bioscience Company), following the manufacturer’s instructions. The monoclonal antibodies specific for mouse TNF-a, IL-6, and IL-1β were aliquoted in 96-well plates. The wells were incubated for 30 min at room temperature and then the test samples were added. The biotin-labelled antibodies against the mouse TNF-a, IL-6, IL-1β and the streptavidin-HRP were added successively. After color development, the absorbance of each well was recorder at 450 nm.

### Statistical analysis

All the data are presented as mean ± SEM from at least three independent experiments. Morris water maze was analyzed with repeated measurements ANOVA. Comparisons between the periodontitis and the control group were evaluated by the Student’s *t*-test. All statistical analyses were performed using the SPSS Statistics software (17.0). Differences with *P* < 0.05 were considered statistically significant.

## Results

### MWM spatial learning and memory tests in young and middle-aged mice

**Fig. 1:**
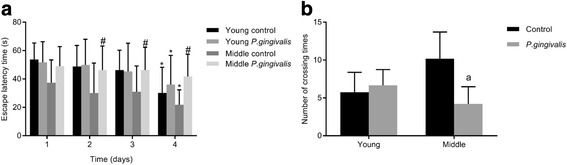
Spatial learning and memory assessed by the MWM test in young and middle-aged mice. (**a**) Escape latency; (**b**) the number of platform crossings. Data presented as mean ± SEM. “*” indicates statistically significant difference (*P* < 0.05) in the escape latency time of mice in the same group, between day 1 and the corresponding day. “#” indicates statistically significant difference (*P* < 0.05) in the escape latency time between *P. gingivalis* infected mice and the corresponding control mice, on the same day. “a” indicates statistically significant difference (*P* < 0.05) in the number of crossing times between *P. gingivalis* infected mice and the corresponding mice control group

During the training days, escape latency was analyzed with repeated measurements ANOVA. The escape latency of middle-aged *P. gingivalis* infected mice was not significantly reduced during the 4 successive days. In contrast, escape latency of the middle-aged mice control group was gradually reduced, especially on day 4 compared to day 1. Both young *P. gingivalis* infected and control mice progressively decreased the time of finding the platform, with the difference being more obvious on day 4 when compared to day 1. There was no significant effect in the escape latency between the young *P. gingivalis* infected and the control mice on day 1. However, the escape latency of the middle-aged *P. gingivalis* infected mice was statistically different from the control group on day 2, day 3 and day 4 (Fig. 1a). During the probe test, no significant variations in the number of crossing times were observed between the young *P. gingivalis* infected and the control mice. As for the middle-aged mice, the *P. gingivalis* infected mice crossed the area where the platform had been located more times than the control group (Fig. 1b). These results clearly indicate that *P. gingivalis* infection can impair the spatial learning and memory abilities of the middle-aged mice. However, *P. gingivalis* infection did not significantly affect the cognitive competence of young mice.

### mRNA expression of TNF-α, IL-6, and IL-1β in brain tissues

**Fig. 2:**
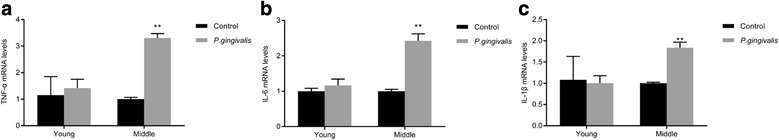
Analysis of the TNF-α, IL-6, IL-1β mRNA expression levels in mice brain tissues by qRT-PCR. (**a**) the mRNA expression of TNF-α in middle-aged *P. gingivalis* infected mice was increased; (**b**) *P. gingivalis* infection increased the mRNA levels of IL-6 in middle-aged mice; (**c**) the mRNA expression levels of IL-1β were increased in middle-aged *P. gingivalis* infected mice when compared to the control group. “**” indicates statistical significant difference (*P* < 0.01) between *P. gingivalis* infected mice and the corresponding control group

It has been revealed that periodontal-induced pro-inflammatory mediators, microorganisms, and products of bacteria can reach the brain through systemic circulation and/or neural pathways and increase the brain cytokine levels [16]. In order to investigate whether *P. gingivalis* periodontal infection could induce inflammatory responses and cause the release of pro-inflammatory cytokines in young and middle-aged mice, we examined the mRNA expression of TNF-α in the brains of mice. We were able to show that the mean mRNA expression levels of TNF-α in the brain tissues of the middle-aged *P. gingivalis* infected mice was increased, in comparison to the control group. However, no significant effect in the mRNA levels of TNF-α in the young mice group was observed (Fig. 2a). Similarly, assessment of other pro-inflammatory cytokines revealed that IL-6 and IL-1β mRNA levels increased only in middle-aged *P. gingivalis* infected mice, but not in young mice (Fig. 2b, c).

### Protein levels of TNF-α, IL-6, and IL-1β in brain tissues

**Fig. 3:**
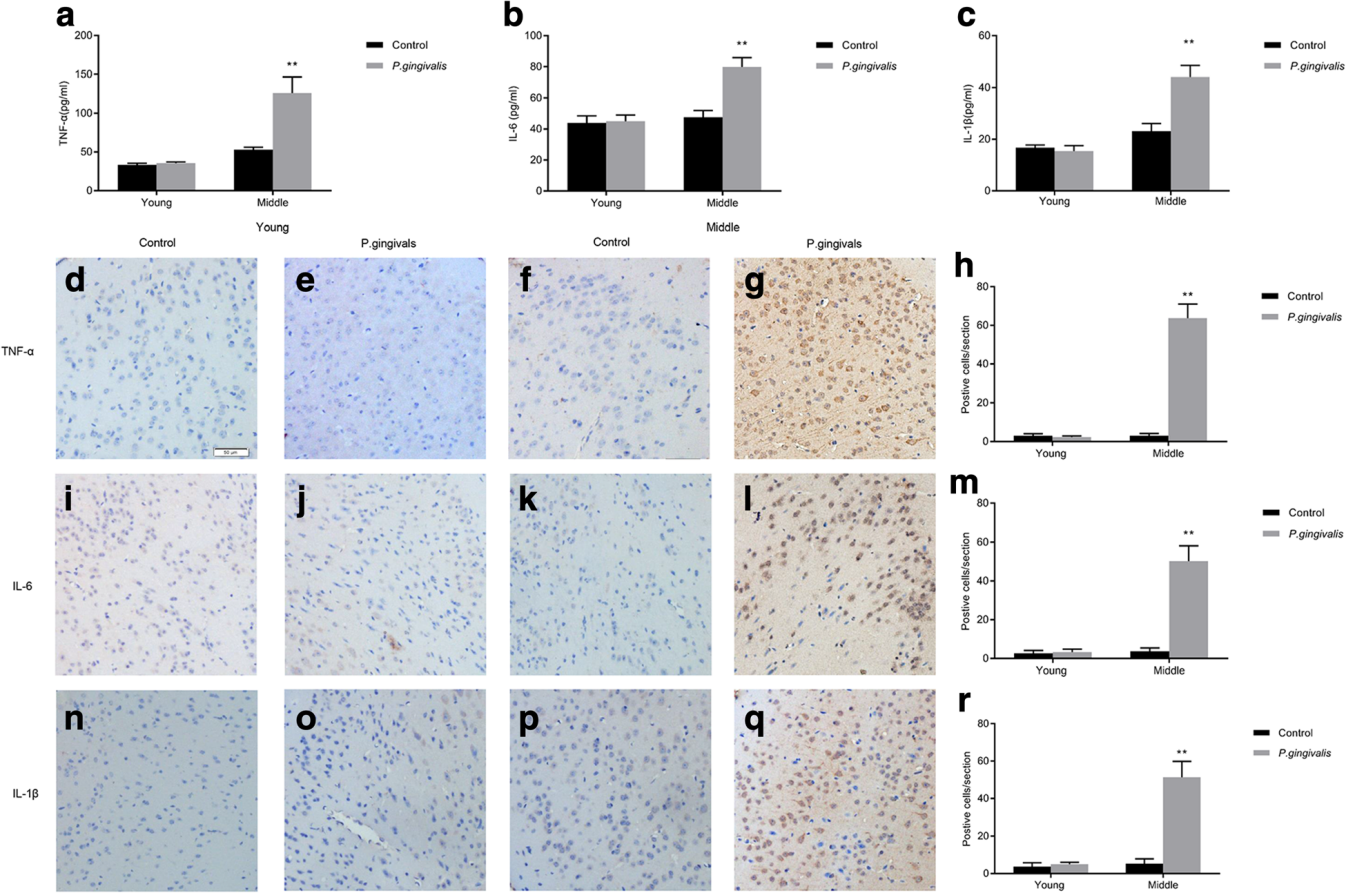
Age-dependent expression of TNF-α, IL-6, IL-1β in mice brain tissues, assessed by ELISA and immunohistochemistry. The protein expression levels of (**a**) TNF-α, (**b**) IL-6, (**c**) IL-1β in brain samples from young and middle-aged mice, measured by ELISA. Immunostaining of mice cerebral cortex tissue samples with antibodies against (**d**-**h**) TNF-α, (**i**-**m**) IL-6, and (**n**-**r**) IL-1β. (**d**, **i**, **n**): young control mice. (**e**, **j**, **o**): young *P. gingivalis* infected mice. (**f**, **k**, **p**): middle-aged control mice. (**g**, **l**, **q**): middle-aged *P. gingivalis* infected mice. Scale bar: 50 μm. “**” indicates statistically significant differences (*P* < 0.01) between the *P. gingivalis* infected mice and the mice in the corresponding control group

We next determined the protein content of TNF-α, IL-6, and IL-1β in the brain tissues. The levels of all of the three pro-inflammatory cytokines were significantly higher in middle-aged *P. gingivalis* infected mice than in control mice. In contrast, the concentration of TNF-α, IL-6, and IL-1β did not differ within the young mice groups (Fig. 3a-c). These findings were also supported by immunohistochemistry analyses. As show in Fig. 3g, the brains of the middle-aged *P. gingivalis* infected mice showed an increased expression of TNF-α compared to the brain tissues from the control group. There was little TNF-α immunoreactivity found in both young *P. gingivalis* infected and control mice group (Fig. 3d, e). Similar to the TNF-α results, IL-6 and IL-1β expression in the *P. gingivalis* infected mice was higher than in the control group of the middle-aged mice. However, IL-6, IL-1β were expressed at low levels, particularly in young mice. These results suggest that *P. gingivalis* infection may promote neuroinflammation by increasing the expression of the pro-inflammatory cytokines TNF-α, IL-6, and IL-1β in the brain tissues of middle-aged mice. Summary tables for statistical analysis are presented in Additional file 1.

## Discussion

The major result of the current study is that *P. gingivalis* infection may cause memory impairment through induced age-dependent neuroinflammatory responses via modulation of pro-inflammatory cytokines release in the middle-aged mice.

Periodontitis is a chronic inflammatory disease, which could induce systemic host responses. Numerous reports have shown that periodontitis could raise the serum pro-inflammatory state, characterized by increased levels of C Reactive Protein (CRP) and pro-inflammatory cytokines (e.g. TNF-α), and decreased levels of anti-inflammatory markers (e.g. IL-10) [17]. Several studies have reported that peripheral inflammation could activate microglia cells and promote the generation of pro-inflammatory cytokines, including IL-1β, IL-6 and TNF-α, in the brain, resulting in neuroinflammation [18]. Neuroinflammation, including activation of microglia cells, participation of astrocytes, and involvement of neurons, has been suggested to contribute to the development of neurodegenerative diseases such as AD, Parkinson’s disease, amyotrophic lateral sclerosis, and multiple sclerosis [14, 15, 19, 20]. Therefore, many studies focus on the correlation between periodontitis and AD. In addition, several reports support the hypothesis that periodontal inflammation can affect cognition. Kamer et al. found that older Danish adults with periodontitis or severe tooth loss exhibit impaired cognition in comparison to healthy subjects [4]. Animal studies have also provided evidence on the impact of tooth loss on neuroinflammation and cognition. Female transgenic mice after tooth extraction were significantly impaired in learning and memory abilities which confirmed that neuronal cell loss in the hippocampus could be triggered by tooth loss causing memory impairment [21]. Although tooth loss can occur for several reasons, periodontitis is one of the major causative factors [22]. Additionally, *P. gingivalis* has been shown to secrete other components associated with periodontitis, which might also affect cognitive function [23]. These results indicate that periodontitis may impair cognition. However, there is limited information on the association between *P. gingivalis* infection and cognition. In the present study, we have observed that *P. gingivalis* infection elevated the expression levels of the pro-inflammatory cytokines TNF- α, IL-6, and IL-1β in the brains of middle-aged mice. It was also noted that middle-aged *P. gingivalis* infected mice displayed impaired learning and memory abilities. These findings provided further evidence for supporting the association between *P. gingivalis* periodontal infection and cognitive impairment.

In order to assess the learning and memory abilities of mice, behavioral tests are required. The MWM test was used to assess learning and memory [24]. It is a maze where the animals must search for a hidden platform which is submerged under the water surface and placed in a fixed location [25]. Our setup is free from motivational stimuli, like deprived food and water, electrical stimuli, and buzzer sounds, which are likely to impact the normal process of memory. Thus, this task is more precise than active/passive avoidance tasks as it eliminates factors which may interfere with the learning and memory like visual acuity and motor function [12, 26]. In general, the MWM is an effective and accurate test for assessing learning and memory abilities. The escape latency data could be interpreted as spatial learning. During the training days, mice spent less time finding the platform, indicating that they have learned and memorized the location of the platform [26]. The number of times of crossing the platform is regarded as the evaluation outcome of the memory assessment of the mice, and a reduction of the crossing times through the platform indicates memory impairment. In our present study, the results showed that the escape latency and the crossing times were statistically significant different between middle-aged mice with *P. gingivalis* periodontal infection and control mice. Therefore, it is possible that *P. gingivalis* periodontal infection promotes age-dependent neuroinflammatory responses though pro-inflammatory cytokines release.

It has been demonstrated that inflammation induces alterations in neurovascular functions, resulting in an increase in the blood–brain barrier permeability, reduction of nutrient supplements, and aggregation of toxins. Elevated levels of pro-inflammatory mediators in the blood, such as IL-β and TNF-α, can lead to their direct or indirect transport to the brain, which might accelerate the development of brain impairment [27]. During the process of neuroinflammation, pro-inflammatory cytokines (TNF-α, IL-6, IL-1β, and others) are essential neuroinflammation mediating signaling molecules [28]. TNF-α has been reported to play a pivotal role in the development and functions of the CNS, including neuron compliance, cognition, and behavior [29]. In the brain, trauma, infection, or the presence of endogenic abnormal protein aggregates, such as amyloid-β (Aβ) peptides in AD, can activate the secretion of TNF-α, primarily produced by glial cells. In addition, TNF-α has been proven to activate immune/glial cells leading to the augment of amyloid-β precursor protein and Aβ deposits in vitro [20]. One study demonstrated that a single intraperitoneal LPS injection in mice caused peripheral TNF-α expression, both at the mRNA and the protein levels, in the brain [30]. As a key molecule, IL-1β is capable of triggering the production of various inflammatory mediators via activating microglia cells. Thus, there is a close correlation between IL-1β levels and neuroinflammation in AD [31]. Some reports have deduced that afferent neurons can respond directly to peripheral cytokines, like IL-1, for vagal sensory nerve activation. IL-1β has been proven to promote the transformation of Aβ from its non-fibrillar form to insoluble Aβ fibers, leading to increase in plaque formation [32]. Concerning IL-6, its up-regulation in the brain of transgenic mice has been shown to be associated with severe neurological dysfunction. Another study using radial arm maze test to examine the spatial learning of mice showed that IL-6 deficient mice exhibit better and faster acquisition of learning and memory abilities [14].

Our approach to study the association between periodontitis and cognition differed from previous studies, as we have used the *P. gingivalis* periodontal infection animal model. We have assessed the effect of the entire *P. gingivalis* on healthy mice, rather than assessing the individual components of *P. gingivalis*. In addition, we directly measured the mRNA and protein levels of TNF-α, IL-1β, and IL-6, in the brains of mice by qRT-PCR and ELISA. We found that the expression of these cytokines showed differences between young and middle-aged mice. Therefore, the effects of aging on neuroinflammation need to be considered. Most studies have used middle-aged or older mice for research purposes. In our study we chose both young and middle-aged mice and found that, unlike the middle-aged animals, the young mice with *P. gingivalis* periodontal infection neither displayed impaired cognition nor overexpressed pro-inflammatory factors. These differences between the young and middle-aged mice might be attributed to chronic inflammation due to aging, which exerts additional stress to the brain nerve cells of older mice and makes them more vulnerable to infection [33]. Moreover, aging-related alterations, like decreased density and plasticity of synapses, and the amount of the pathological neurofibrillary tangle formation required to cause dementia, increase with age [34, 35]. Additionally, during systemic inflammation, the functions of the blood-cerebrospinal fluid barrier (BCSFB) were significantly affected by the differential responses of glial cells to age-dependent cytokines [36]. Recent reports showed that chronic systemic inflammatory processes promoted the transformation of microglia and astrocytes into anti-inflammatory cell types in young rats, while a pro-inflammatory cell phenotype was detected in middle-aged rats [37]. Furthermore, aging is the major risk factor of AD and is correlated with elevated glial responsiveness, which might increase the brain’s susceptibility to injury and disease [21, 38]. One study supports this view by showing that the age-associated progression of the AD-like phenotype in WT mice could be initiated by chronic inflammatory conditions with enhanced accumulations of APP [39].

The present study has some limitations. First, although over-activation of microglia has been reported to be a hallmark of neuroinflammation [40], we did not investigate the activation of microglia in our study. However, the objective of this paper was to determine whether *P. gingivalis* periodontal infection can promote cognitive impairment via inducing neuroinflammation, which was assessed by measuring the levels of the main pro-inflammatory cytokines. Second, the brain inflammation induced by *P. gingivalis* periodontal infection may be mediated via the systemic circulation and/or direct neural pathways. In this study we did not access which mediating pathway is involved, which needs to be considered in the future. In addition, although TNF-α, IL-6, and IL-1β are the most reported molecules to be implicated in neuroinflammation, they cannot represent the whole range of inflammatory cytokines. Therefore, other inflammatory cytokines associated with neuroinflammation remain to be considered.

## Conclusion

We have shown that *P. gingivalis* periodontal infection may induce an age-dependent brain inflammation, which increases the TNF- α, IL-6, and IL-1 β levels in middle -aged mice. Moreover, the middle-aged *P. gingivalis* infected mice displayed impaired cognition. Although we have not demonstrated that periodontitis is a risk factor for AD, these findings showed that *P. gingivalis* periodontal infection can cause memory impairment, which suggests that periodontitis might have a similar effect on the development of AD.

## Additional file

Additional file 1: **Table 1.** Escape latency time(s) assessed by the MWM test in young and middle-aged mice. **Table 2.** The number of platform crossings assessed by the MWM test. **Table 3.** Analysis of the TNF-α, IL-6, IL-1β mRNA expression levels in mice brain tissues by qRT-PCR. **Table 4.** The protein expression levels of TNF-α, IL-6, IL-1β in brain samples, measured by ELISA. **Table 5.** The mean number of the TNF-α, IL-6, IL-1β positive cells per section. (XLSX 14 kb)

## Abbreviations

AD: Alzheimer’s disease
Aβ: Amyloid-β
BCSFB: Blood-cerebrospinal fluid barrier
CMC: Carboxymethyl cellulose
CNS: Central nervous system
CPR: C Reactive Protein
LPS: Lipopolysaccharide
MWM: Morris water maze
*P. gingivalis*: *Porphyromonas gingivalis*
PBS: Phosphate buffer saline
